# Simultaneous Pancreas-Kidney Transplant: A Positive Stimulus in the Medical World

**DOI:** 10.7759/cureus.8308

**Published:** 2020-05-27

**Authors:** Muna A Ali, Safeera Khan

**Affiliations:** 1 Nephrology, California Institute of Behavioral Neurosciences & Psychology, Fairfield, USA; 2 Internal Medicine, Apollo Health City, Hyderabad, IND; 3 Internal Medicine, California Institute of Behavioral Neurosciences & Psychology, Fairfield, USA

**Keywords:** treatment options for diabetic nephropathy, esrd and management, economic drawbacks of transplant, spkt and its effect in recent years

## Abstract

Kidneys are one of the essential organs of our body, with chronic kidney disease being a very prevalent and emotionally, mentally and physically straining condition affecting 1 in 15 people worldwide. The prevalence is further escalating with every passing year. It is slowly progressive in nature, and many times goes unnoticed until symptoms start manifesting and presenting themselves much later in life. In this article, end-stage renal disease (ESRD) due to diabetes mellitus and its effect on different organs is examined, along with the role of simultaneous pancreas-kidney transplant (SPKT) in the management of this condition. Although proven to be an assured treatment with an outstanding allograft acceptance rate, the fact that it is still not widely adopted in many healthcare setups due to financial implications is also studied.

Online databases such as PubMed and Google Scholar were searched for the purpose of data collection; due to the very limited number of randomized controlled trials conducted on this given topic, a limited discussion was retrieved. By applying the Preferred Reporting Items for Systematic Reviews and Meta-Analyses (PRISMA) method and several inclusion/exclusion criteria, approximately 66 articles were assessed for eligibility based on the title and abstract. A total of 44 articles were shortlisted and considered in the final review.

Several systematic reviews that have been conducted in the past reveal the importance of SPKT at an early stage of diagnosis towards increasing longevity of the patient with freedom from multiple medications. Transplant is a cost-effective therapy when compared to the prolonged dependence on dialysis, insulin pens, and increased susceptibility to infections.

A greater number of specialists must also train to carry out SPKT and identify the early stages of ESRD, and medical centers should be encouraged to carry out transplant procedures effectively both financially and medically. This can be achieved through the development of global policy mechanisms and establishment of universally adoptable standards.

## Introduction and background

“Scientific medicine is one of the greatest triumphs of humankind.” - Raymond Tallis.

End-stage renal disease (ESRD) is most often caused by chronic illnesses, and diabetes mellitus (DM) is one of the major contributors to nephropathy. Modern science has evolved with time and introduced many treatment modalities to help avoid further complications and to decrease mortality over a period of time. One such addition is simultaneous pancreas-kidney transplant (SPKT), which is "the next big thing" in the world of organ transplants.

The intended goal of a successful SPK transplantation is that the patient not only recovers from uremia but also achieves lower or normal blood glucose levels and can maintain control without the use of insulin or other hypoglycemic agents. Accordingly, this combined procedure has become an established solution for type 1 diabetic patients with ESRD increasing short-term morbidity and mortality risk [1]. Priority should be given to patients who would need dual requirements. The patient does not have to rely on hemodialysis further and has the freedom of mobility while simultaneously, the procedure prevents the periodic attack of diabetic nephropathy in a renal allograft.

Chronic hemodialysis/peritoneal dialysis have their setbacks, with sepsis and intradialytic hypo- and hypertension being the most prevalent complications with a high likelihood. Diabetic neuropathy is only reversible with a transplant, as the middle molecules (size 500-20,000 Da) fail to filter on routine dialysis, leading to dependence on neuropathic agents and also an increase in monthly costs of total medicines. These inflated costs, including that of dialysis, create a financial burden on patients and their families. Appropriate post-transplant care and management with maintenance therapy (steroid + mycophenolate mofetil + tacrolimus or cyclosporine) and routine follow-ups to assess the prognosis will be beneficial.

In this article, a hypothetical case study of a patient with ESRD, who is on dialysis and developed calciphylaxis of both lower limbs, is discussed [2]. Also, despite being a popular management option, the current declining trend of SPKT in modern healthcare, the future prospects, and financial implications of SPKT will be discussed.

Case report (hypothetical)

A 38-year-old Indian origin man is evaluated for painful skin lesions that have developed over his lower limbs during the past one week. He has a history of ESRD due to long-standing hypertensive and diabetic nephropathy, for which he undergoes hemodialysis three times a week. Upon physical examination, the patient is a well-developed, well-nourished individual with a blood pressure of 170/100 mm Hg, pulse rate 84, respiratory rate 24, temperature 37°C, and bodyweight of 80.5 kg. The cardiovascular exam was unremarkable; respiratory system examination showed positive bilateral air entry, with mild crepts present bilaterally. HEENT was remarkable for fundoscopic findings of retinal hemorrhages and cotton wool patches consistent with the hypertensive injury.

The rest of the physical examination showed grade 2 edema on both lower limbs and superficial excoriation of his skin as a result of scratching. Tables 1, 2 show the lab values obtained.

**Table 1:** Laboratory data

Chemistry	Results	Normal values
Hemoglobin	09	13-18 g/dl
Hematocrit	27.4	40-54%
Mean corpuscular volume	88	85-95 FL
Serum alkaline phosphatase level	310	30-110 IU/l
Serum parathyroid hormone	700	10-65 pg/ml
Serum creatinine	15	0.7-1.5 mg/dl
Serum glucose	110	70-110 mg/dl
Serum calcium	07	8.9-10.3 mg/dl
Serum phosphorus	12.5	2.6-6.4 mg/dl
Serum sodium	132	136-146 mmol/l
Serum potassium	6.9	3.5-5.3 mmol/l
Serum carbon dioxide	15	23-27 mmol/l
Serum chloride	105	98-108 mmol/l
Blood urea nitrogen	150	7-22 mg/dl

**Table 2:** Urine analysis

Urine routine (complete urinary examination)	Results
pH	06
Specific gravity	1.010
Protein	3+
Glucose	Positive
Acetone	Positive
Occult blood	Large
Bile	Negative

The 800-ml 24-hour urine sample showed protein levels at 600 mg/dl and creatinine at 180 mg/dl. The renal ultrasound report suggested that the right kidney measured at 8 x 6 cm and left kidney at 8.2 x 5.8 cm. Both kidneys illustrated hyperechogenecity while hydronephrosis was not seen.

## Review

Methods

**Figure 1:**
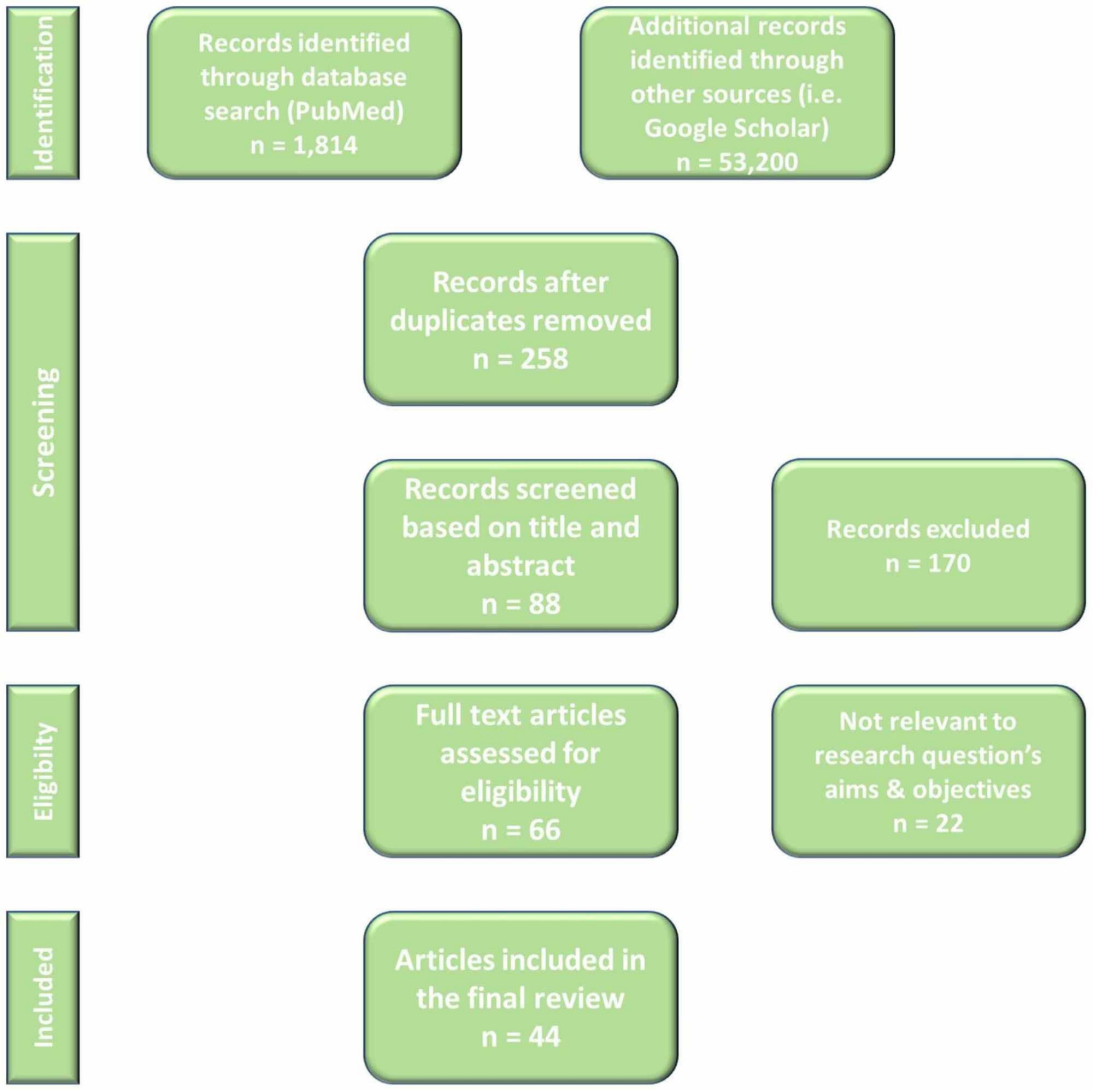
PRISMA approach for selecting studies PRISMA, Preferred Reporting Items for Systematic Reviews and Meta-Analyses

We searched online databases PubMed and Google Scholar for data collection. Due to the very limited number of randomized controlled trials (RCTs) done on this given topic, a limited discussion was retrieved, and so all types of studies were chosen. By applying the Preferred Reporting Items for Systematic Reviews and Meta-Analyses (PRISMA) method, several inclusion/exclusion criteria were applied. Inclusion involved gathering papers from the last 10 years that were relevant to the topic and published. Animal studies were excluded. As shown in Figure 1, approximately 66 articles were assessed for eligibility based upon the title and abstract. A total of 44 articles were shortlisted and considered in the final review.

Results

The eight studies listed in Table 3 were selected out of the total of 44 studies that were reviewed due to their strong relevance to the research objectives of this article. The shortlisted studies varied and contrasted in their methodologies and findings, but they allowed for a comparative analysis that led to the identification of limitations in their respective conclusions. Although the collective aim of these studies was undoubtedly to identify means to reduce patient hardship and suffering through potential advancements in treatment options, this review aims to summarize their findings and present a better alternative.

**Table 3:** Summary of studies ESRD, end-stage renal disease; DM, diabetes mellitus; GVHD, graft versus host disease; LDKT, live donor kidney transplant; LDRT, live donor renal transplant; PAK, pancreas after kidney; QOL, quality of life

Study	Study type	Purpose of study	Conclusion
Weng et al. [3]	Randomized control trial	Evaluated knowledge on LDKT	Before the procedure, it is mandatory to explain the benefits, risks, and the type of transplant to the recipient. The study reveals a lack of awareness among recipients on the LDKT procedure and its benefits over other transplant options.
Rashidi et al. [4]	Literature review, observational study	Evaluated chimerism post-SPKT using fluorescence in situ hybridisation for sex chromosomes	The exact pathology involved in GVHD after SPKT is still unknown, though rarely occurs in this case. Recommended for collaboration between the stem cell transplant team and organ transplant team, to find out reasons behind GVHD and find answers to minimize complications.
Chan et al. [5]	Systematic review	SPKT benefits for DM type 1 and 2 with ESRD	According to this study, SPKT should only be done after all other conservative efforts have failed in managing hyperglycemia, as a last resort, given that it is a high-risk procedure with satisfactory results in controlling disease, graft acceptance, and improving QOL.
Fridell and Powelson [6]	Systematic review	Advantages of PAK transplant and its nominal popularity compared to other transplants	LDRT remains the most popular while SPKT is ideal for DM type 1 and 2 with ESRD. PAK transplant is acceptable with improvement in post-transplant immunotherapy, thereby leading to reduced chances of allograft rejection. This should be considered in patients who have already undergone renal transplant to control diabetes.
Redfield et al. [7]	Observational study	Current trends and future prospects of SPKT in comparison with pancreas transplant	The popularity of pancreatic transplant has decreased due to improvements in surgical techniques and preference for SPKT. Given the half-life, post-SPKT, the pancreatic graft survival has steadily progressed to 14 years. Also, late steroid withdrawal with caution is a major concern to make the transplant a success. Future surgical techniques must be encouraged to have advances in steroid-free immunosuppression therapy and immune monitoring.
Wiseman [8]	Systematic review	To promote benefits Of pancreatic transplant In diabetic ESRD patients	Subjective assessment of donor organ quality, improvement in standardization, and transplant protocols must be put in place for growth in pancreatic transplantation. Patients must be explained the advantage of various pancreatic and kidney transplant over chronic dialysis.
Ganji et al. [9]	Observational study	To evaluate financial awareness among individuals with regard to pre- and post-transplant	The study revealed a lack of awareness and knowledge about costs involved in both pre-transplant and post-transplant procedures. Not enough details are provided about available health insurance policies and resources made available for their aid. This gap must be addressed, and policies must be affordable to facilitate such individuals.
Myint et al. [10]	Observational study	Compare QOL in patients pre-transplant vs on dialysis with multiple comorbidities and post-pancreatic or SPKT	The study revealed that patients undergo immense emotional, physical, and psychological stress during both pre- and post-SPK transplant, but their scores were low when compared to other patients with comorbidities and on dialysis. Most of the younger population though didn’t report any difference but were happy with improved QOL and were fit. Increased targeted support must be provided to enable them to overcome challenges involved in treatment plans.

The shortlisted studies further proved that significant awareness is required among patients and the donor, to consider SPKT. Also, it was observed that SPKT promotes enhancements in the mental and emotional well-being of the patient, as discussed below. Several systematic reviews that have been conducted in the past reveal the importance of SPKT at an early stage of diagnosis towards increasing longevity of the patient with freedom from multiple medications. Observational studies help obtain a clear picture of the future trends associated with the procedure. Transplant is a cost-effective therapy when compared to prolonged dependence on dialysis and insulin pens, with increased susceptibility to infections.

Discussion

As the millennial population grows at an exponential rate, so is the advancement of the healthcare system, intending to provide the best care and management of diseases. Shifting our focus from the prolongation of life expectancy to improving the quality of life to be lived, this mere ambition of healthcare providers requires massive levels of encouragement, motivation, and cost-effective care to help the less fortunate without compromising on service quality.

**Figure 2:**
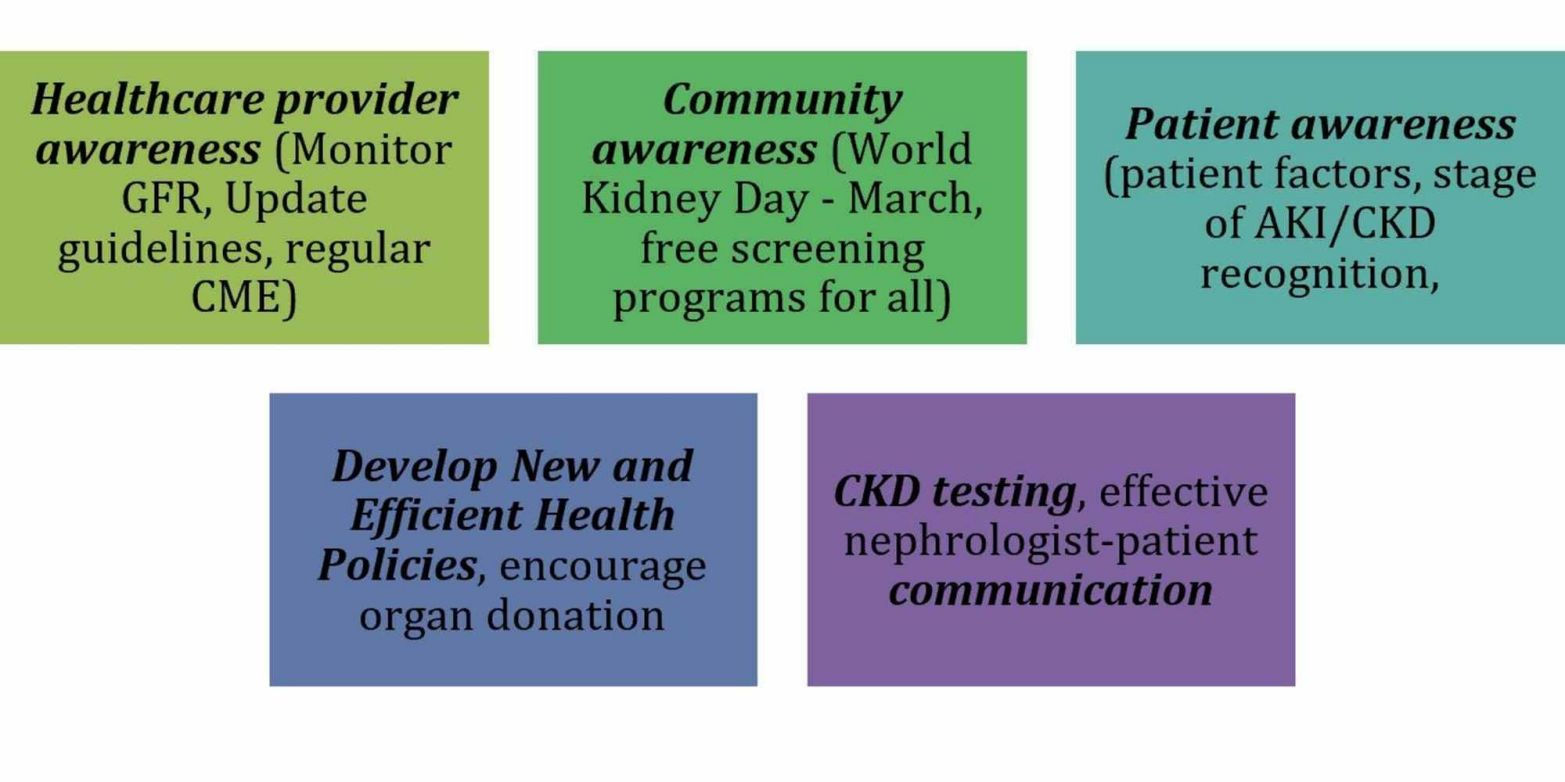
Depiction of prevention and management at various levels AKI, acute kidney injury; CKD, chronic kidney disease; CME, continuing medical education; GFR, glomerular filtration rate

Kidneys are essential to wash out all the toxins from the body. However, many factors are possibly associated with a lack of awareness among people, which would help guide the implementation of awareness efforts that are yet to be fully examined. These factors not only help to improve clinical outcomes but also decrease the prevalence of chronic kidney disease (CKD) [11]. Figure 2 represents prevention and management solutions at various levels.

Management of ESRD

The above-mentioned case report of a diabetic ESRD patient with calciphylaxis, already undergoing hemodialysis, needs further management. The need for SPKT can be explained to the patient and his family along with its benefits.

ESRD or failure is the inability of the kidneys to function properly. It is an irreversible damage, ultimately leading to hyperkalemia and pulmonary edema over days to weeks. If not corrected timely, this may eventually be fatal, with the estimated glomerular filtration rate reaching stage 5 at <15 ml/min/1.73 m^2^ [12].

One of the best solutions to this stated problem is undergoing a renal transplant (for suitable patients), which is one of the most commonly transplanted organs in the world, along with a pancreatic transplant. According to Lambers Heerspink and de Zeeuw (2011), "the mortality rate of patients with diabetic nephropathy is higher than the average mortality rate of all types of cancer" [13]. Such patients who are suffering from uncontrolled diabetes, which eventually leads to complications like nephropathy, retinopathy, or neuropathy, are often treated on dialysis and insulin or oral hypoglycemic agents and are suggested to undergo either pancreas transplant alone (PTA) or SPKT.

During the period between December 1986 and April 1988, a pancreaticoduodenectomy (Whipple procedure) with simultaneous renal transplant done in an RCT was successfully undertaken for the first time ever. With successful post-operative therapy, the patient's mean serum creatinine of 1.75 mg/dl was achieved. The recipient group was of a young-age population, with only 10 individuals requiring extended hospital stay post-procedure for further management. But this gave hope and opportunity to perform this combination surgery as a definite treatment for ESRD with DM [6].

The idea of encouraging pancreatic transplant started in 1993, and the outcome alleviated excellent results. This, when combined with the renal transplant, ascertains to be a cost-effective and life-saving therapy. According to an overview of the International Pancreas Registry (Gruessner 2011), "the types of pancreas transplantations performed have been simultaneous with a kidney (SPK) (75%), those given after a previous kidney transplantation (PAK) (18%), and pancreas transplantation alone (PTA) (7%)" [13]. Over the years, optimization of surgical techniques and low-toxicity immunosuppression have slowly enhanced the outcomes of pancreas transplantation, considering patient survival and graft function. Patient survival now reaches over 95% at one-year post-transplant and over 83% after five years. The best graft survival was found in SPK with 86% pancreas and 93% kidney graft function at one year. For pancreas after kidney (PAK) transplants, the pancreatic graft function was 80%, and for PTA, the pancreatic graft function reached 78% at one year [13].

Calcific Uremic Arteriolopathy

Calciphylaxis is a fatal disorder in which gradual calcium salt deposition occurs in vessels of immunocompromised individuals who are suffering from chronic renal failure and are on dialysis; it is very rarely seen in patients with no renal involvement. Clinically, patients present with non-healing ulcers or plaques on the lower limbs resembling cellulitis leading to gangrene and infection; therefore, diagnosis is routinely missed by Internists [14].

Its pathology is still unknown; most researchers have found some key elements elevated along with uremia, such as high calcium phosphate and parathyroid hormone levels, vitamin D elevation or excessive intake of it, imbalance in promoters and inhibitors of calcification, uncontrolled DM, triglycerides, and hypertension - all of which may contribute to the formation of calcified plaques in vessels and on skin surface deposits. Performing a skin biopsy may also assist in reaching a diagnosis.

This can be treated with intense dialysis, prescribing parathyroid analogs, introducing phosphate binders, ruling out secondary infections, and prescribing suitable antibiotics if the need arises. If all fail to reduce the elevated hormone and electrolytes, then patients should be referred for for parathyroidectomy, and, eventually, SPKT, which is a definitive solution.

Side Effects of Long-Term Dialysis

**Figure 3:**
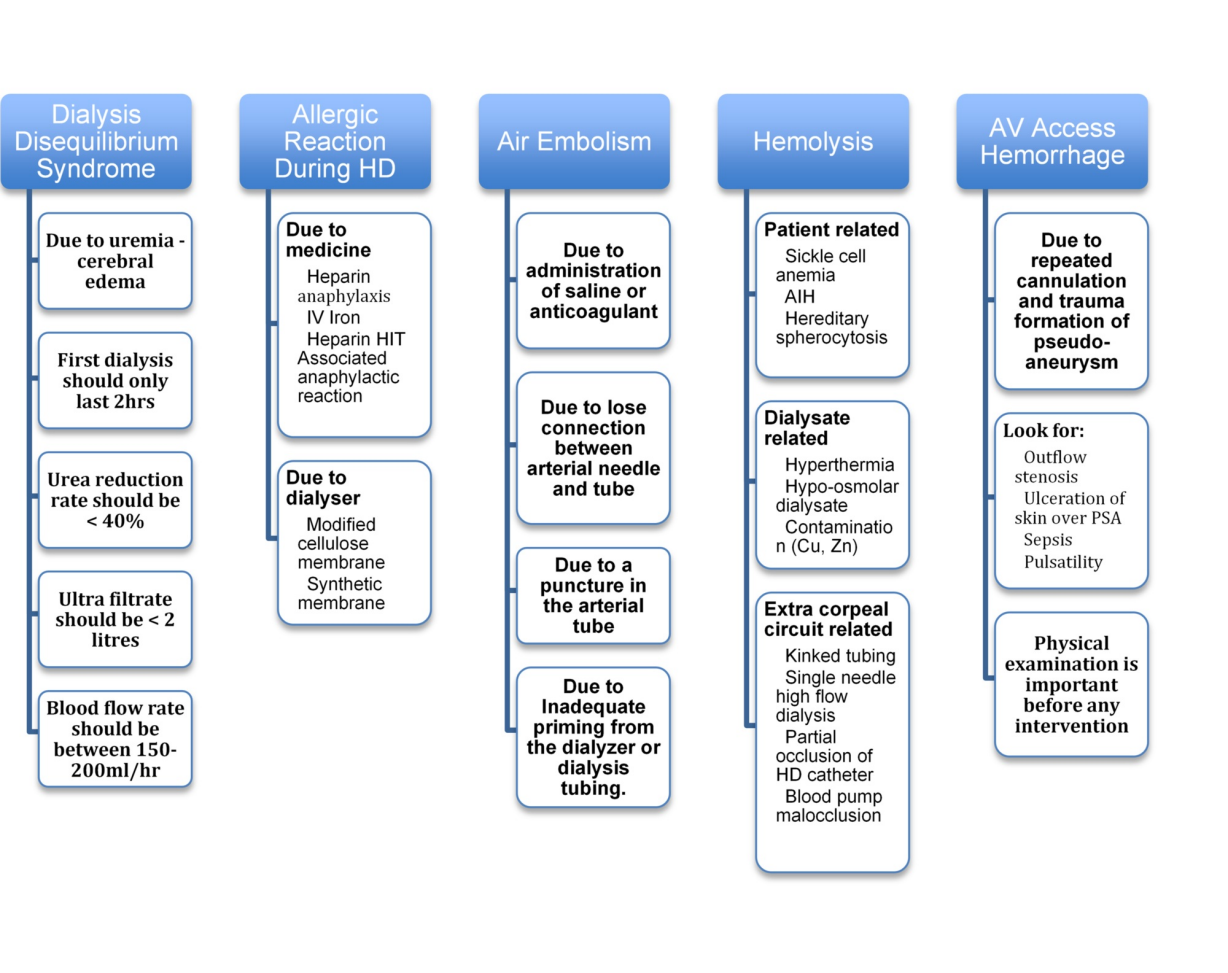
Side effects of long-term dialysis HD, hemodialysis; HIT, heparin-induced thrombocytopenia; AIH, autoimmune hepatitis; PsA, psoriatic arthritis

**Figure 4:**
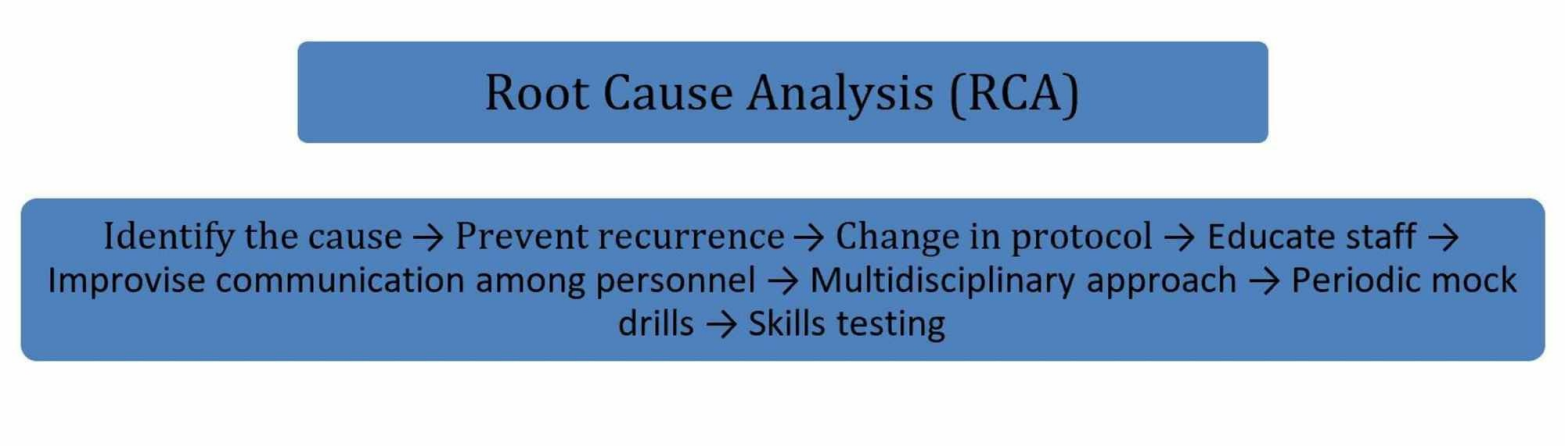
Illustrative breakdown of RCA

Most chronic kidney diseases are irreversible. Once the patient reaches end-stage renal failure, dialysis is the only way out. Dialysis is life-saving but lifelong until a kidney transplant is done along with some lifestyle transformations [15]. Figures 3, 4 represent long-term side effects of chronic dialysis and root cause analysis.

Various Types of Transplants

PAK and SPK have increased chances of mortality as the former requires two sequential surgeries, and the latter in itself is a major surgical procedure. However, both tend to offer improvement in the quality of life. PAK has been linked to superior rejection in pancreas allograft in comparison with SPK [16]. Islets of pancreas transplantation is an impressive alternative in discussion for people with diabetes without any renal involvement. Though not much data was available on this, it definitely could make a positive impact if post-transplant maintenance therapy is successful as well, making them independent of insulin administration. The characteristics of various types of transplants are highlighted in Table 4.

**Table 4:** Characteristics of various types of transplants DDKA, deceased donor kidney allograft; LDKA, live donor kidney allograft; PAK, pancreas after kidney; SPK, simultaneous pancreas-kidney

	Types of transplants			
	DDKA	LDKA	PAK	SPK
Waiting period	Longer	Shorter	Longer	Longer
Glucose control	Nil	Nil	Excellent	Excellent
Mortality rate	Low	Moderate	Moderate	High

Financial Implications for SPKT and Other Concerns

Several studies have been carried out in the past to assess the economic shortcomings in transplant patients - both pre- and post-procedure - to identify poor transplant outcomes and lack of patient awareness on available financial guidance prior to the procedure [7,17-21]. Another research conducted to study the shortcomings of economic drawbacks in transplant procedures revealed many loopholes and mental stress points for patients and their families; most were not aware of the challenges associated with changes in health insurance policies if a patient develops ESRD or the lack of financial assistance for living donors. Furthermore, no proper guidance for the various transplant procedure options was made available [9].

All of these issues can be well addressed if a global transplant policy and universal health coverage are introduced at affordable packages, and different types of renal transplant options are made available as per patient requirements. Early transplant referral must be done to help decrease the chances of post-transplant infections and graft rejection. Specialized medical oversight must be assigned to advise and effectively educate the family and patient accordingly about pre- and post-transplant costs and also to address other concerns, as well as to ensure that all financial implications are conveyed.

Living organ donations must be encouraged to decrease the waiting list period for transplant recipients, and the donors must not be discouraged by firms or other workplaces from contributing to the wellbeing of the community and themselves. There must be a paid leave break given to live donors to help them volunteer for a noble cause without having to worry about losing their job or loss of pay. Compared to deceased donor kidney allograft (DDKA), live donor kidney allograft (LDKA) is much healthier, more affordable, requires shorter cold ischemia time, and offers better allograft function. Presently, the DDKA waiting list ranges from three to eight years, while the LDKA waiting list ranges from six months to one year.

With the growing health concerns, regular health checkups among family members must be advised to detect and prevent any further disease progression, especially among those who are suffering from DM or adult polycystic kidney disease. Remarkably, kidney donors survive longer than the average individual [22,23].

Overcoming the Decreasing Trend of SPKT

This is one of the most promising treatment options available to patients. Despite promising outcomes, it has not been carried out frequently. There are multiple reasons as to why its popularity has not grown; some of these reasons are positive while others portray a negative contextual background. It has long been perceived that due to the current approach, the deserving candidates fit for this type of surgery are not guided towards specialist centers offering SPKT. The process of donor screening has also become far more stringent, thereby leading to a decrease in the general availability of suitable organs. This explains the current decreasing trend, which implies a sense of quality versus quantity.

However, on a positive note, diabetic education has led to the prevention of further disease progression, and a growing trend in well-controlled DM through insulin has also led to a decline in pancreatic transplants among the older generation compared to the younger generation [23]. As it's a known fact that the pancreatic allograft acts as a nephroprotective agent, there are minimal chances of a successive decline in renal function and decreased opportunities for a recurrent hyperglycemic attack.

Limitations of the Review

There were several limitations to the completion of this study. During the search for various articles pertaining to SPKT, information was significantly limited as not many RCTs have been done in the past on this subject, even though it is a successful management option with an excellent outcome and patient satisfaction along with the improved quality of life. Additionally, articles published prior to five years ago were also included in the survey along with a few articles of foreign language origin, as resources were quite limited for the purpose of this study. Also, animal studies were excluded from this study.

Therefore, the exact reasons of less efforts to encourage patients towards undergoing this procedure were not specifically identified, which may also be due to significant delays in diagnosis and early referrals. Furthermore, there are limited numbers of special centers within reach for many deserving candidates. Finally, policies have not been cultivated or developed to help patients financially cover the expenses of such surgeries.

## Conclusions

Conclusively, the role of SPKT in ESRD management has been studied, and it was observed that patients will benefit from this approach to treatment by minimizing use of insulin or other hypoglycemic agents. Several systematic reviews were examined to conclude that the adoption of SPKT is critical towards achieving freedom from multiple medications, and transplant is a cost-effective therapy when compared to dialysis. Although financial implications exist, patients can approach this treatment in greater numbers through awareness programs, regular screening (particularly for high-risk patients, such as the Kidney Early Evaluation Program [KEEP]), financial support programs, and improved self-management strategies. Additional studies are required to understand factors and consequences associated with the lack of guidelines relating to SPKT and to discourage patients from relying on dialysis, given the option to avail a better treatment via transplant.

Healthcare policies targeting reduction of waiting times and enhanced nephrologist awareness will result in increased capacity of the healthcare system. Tied with public awareness programs aimed at donors, availability of donor organs can be increased along with a reduction in waiting lists. Simultaneously, increasing patient awareness will result in greater donor participation. As healthcare providers increase capacity and donors increase availability, the rising demand from patients will be met with a drop in waiting times and increase in affordability. This article is intended to promote awareness among providers and patients alike, which is essential as it addresses gaps in the current approach, and aims to form a bridge between providers and patients, and also aims to raise awareness of the shortcomings in the current treatments pursued by healthcare providers.
